# A comparative gene expression database for invertebrates

**DOI:** 10.1186/2041-9139-2-17

**Published:** 2011-08-24

**Authors:** Mattias Ormestad, Mark Q Martindale, Eric Röttinger

**Affiliations:** 1 Kewalo Marine Laboratory, PBRC, University of Hawaii at Manoa, 41, Ahui Street, Honolulu, 96734, HI, USA

## Abstract

**Background:**

As whole genome and transcriptome sequencing gets cheaper and faster, a great number of 'exotic' animal models are emerging, rapidly adding valuable data to the ever-expanding Evo-Devo field. All these new organisms serve as a fantastic resource for the research community, but the sheer amount of data, some published, some not, makes detailed comparison of gene expression patterns very difficult to summarize - a problem sometimes even noticeable within a single lab. The need to merge existing data with new information in an organized manner that is publicly available to the research community is now more necessary than ever.

**Description:**

In order to offer a homogenous way of storing and handling gene expression patterns from a variety of organisms, we have developed the first web-based comparative gene expression database for invertebrates that allows species-specific as well as cross-species gene expression comparisons. The database can be queried by gene name, developmental stage and/or expression domains.

**Conclusions:**

This database provides a unique tool for the Evo-Devo research community that allows the retrieval, analysis and comparison of gene expression patterns within or among species. In addition, this database enables a quick identification of putative syn-expression groups that can be used to initiate, among other things, gene regulatory network (GRN) projects.

## Background

Laboratories which use well-established developmental biology model systems have recognized the importance of species-specific databases and developed extensive tools for their community, for example Zfin (zebrafish, http://zfin.org) [1], MEPD (medaka, http://ani.embl.de:8080/mepd/) [2], FlyBase (*Drosophila*, http://flybase.org[3], BDGB (*Drosophila*, http://www.fruitfly.org/DGC/index.html) [4], WormBase (*C. elegans*, http://wormbase.org), Aniseed (*Ciona*, http://aniseed-ibdm.univ-mrs.fr/), Gene Expression Database (GXD) at MGI (mouse, http://www.informatics.jax.org/) [5-7]) EMAGE (mouse, http://www.emouseatlas.org) ([8-10]), XENBASE (*Xenopus*, http://www.xenbase.org) ([11-13]) and a comparative database 4DXpress for major animal model species http://ani.embl.de/4DXpress[14]. Recently, efforts have also been made to create databases for emerging model systems such as cnidarians (StellaBase (http://www.stellabase.org) [15] and *Platynereis *PEPD (http://ani.embl.de:8080/pepd/). These databases are mainly used to combine all available resources (genome, expressed sequence tag's (EST), transgenes, publications, expression data, and so on) and the large amount of data makes it sometimes tricky to retrieve a simple gene expression pattern and its related information in an intuitive manner.

Although some of these databases offer comparison between taxonomically related species (for example, among tunicates, Aniseed), we know of no user-friendly and intuitive tool for large-scale comparison of gene expression patterns among diverse organisms.

To address questions about functional embryonic development and body plan patterning or evolution, it is crucial to identify genes involved in developmental processes and tissue specific markers.

The increased feasibility of sequencing projects, due to massively parallel sequencing technologies, is leading to a steady appearance of new data and gene expression patterns from a growing list of formerly understudied species. Currently, comparison of gene expression patterns among species involves numerous hours of searching for the publications of interest. Interpretation of the data may be quite difficult in the case of taxonomically unrelated organisms. In order to collect, manage and facilitate the process of organization and mining of data we have created a freely accessible online comparative gene expression database for invertebrates. We expect this to aid in the study, analysis and interpretation of developmentally regulated processes across evolutionary diverse organisms. The following sections will describe the basic features of that database and how to easily identify putative syn-expression groups [16] for further GRN analysis. A more detailed manual of this scientific tool can be downloaded directly from the database website (http://www.kahikai.org/index.php?content=genes).

### A comparative gene expression database for invertebrates

As several genomic and/or transcriptome resources are already available for most currently studied species (vertebrate as well as invertebrate models), we focused our interest on an intuitive way to gather, store, make available and compare gene expression patterns of invertebrates. Currently, the database contains data from 18 different species from seven distinct phyla (Ctenophores, Cnidaria, Aceolomorpha, Ecdysozoa, Lophotrochozoa, Echinodermata, Hemichordata), more than 180 genes, 210 experiments (72 'unpublished', that is not accessible to the whole community) and more than 1,300 images. The database is used for storing and browsing *in situ *gene expression data as well as immunohistochemistry (IHC) information, comparing gene expression patterns within or among species and identifying possible syn-expression groups. The contributor can assign expression data to different experiment types such as wild type expression, drug treatments, mRNA or morpholino injection and so on, providing an easy means of organizing functional studies. The unique aspect of this database is the flexibility of data storage and organization allowing for a set of gene expression patterns whose access can be distributed to a given laboratory, a group of collaborators or the whole community, by the contributor (refer to the online manual for more details). In any case, the data will always be accessible to the current members of a laboratory who can add new experiments to complete missing information (for example stages) or complementary information (for example drug treatments, gene perturbation) for a given expression pattern. This database can be accessed from anywhere in the world and is backed up frequently on the host servers http://www.dreamhost.com, removing this burden from individual laboratories.

### Construction and data integration

The primary objective of this platform is to create one database in which users can easily compare datasets from a diverse selection of organisms. In this first version of the database we have chosen not to include any sequence information and instead fully concentrate on the comparison of manually annotated expression patterns. Since these kinds of comparisons are fairly simple we have chosen to build the site using a PHP/MySQL backend. The manual annotation steps are kept to a minimum in order to keep data uploads homogenous and straightforward for the user. Our database can store all the information required for the MISFISHIE standard (minimum information specification for *in situ *hybridization and immunohistochemistry (IHC) experiments) ([17,,18]). This format will allow us to integrate other systems and provide the information required to interact and exchange information with other resources in the future.

### Integration of this database into an online community

This database is openly accessible via Internet and is hosted on our web community platform 'Kahi Kai' (meaning 'one ocean' in Hawaiian, http://www.kahikai.org), allowing researchers to interact, collaborate, and share their data. All contributions will have a clear identification of the author of the expression pattern and the laboratory they are associated with. Every user can decide if they want to 'publish' and therefore share the gene expression pattern with the whole community or keep it 'unpublished' and visible only to a user-defined subset of members (for example the lab where he/she works). These 'unpublished' expression patterns can be made accessible to other groups, allowing collaboration and sharing of unpublished data with other users outside of the local lab (refer to the online manual). For security reasons and to avoid unsolicited content in our database, unpublished data are also visible to the site administrators and will be handled with the highest confidentiality. Our vision is that this database becomes an integral part of the Evo-Devo community and that it will provide a useful tool for ongoing and future collaborations.

### Expression data

#### Gene orthology

The classification of genes from multiple organisms into orthologous groups is the prerequisite for comparing gene expression and function. Although approaches to identify gene orthologies have improved in the last decade(s), concerns still exist especially in non-bilaterian animals such as cnidarians, sponges or ctenophores, in which orthology can be problematic and complete annotated genomes that are required to identify paralogy groups, may not yet be available. Therefore, we decided not to implement an automatic way to analyze gene orthologies in the first version of this database, but plan to do so when a reliable online platform becomes available. In the meantime each user has responsibility for assigning gene orthology before publishing it within the database (as is the case for peer reviewed articles). All genes that cannot be assigned to a clear ortholog group can be named as XXX-like gene (for example. 'nodal-like'). Raw sequence data will be available as a Genbank retrieval accession number.

#### Developmental stages and expression domains

Comparing developmental stages within a single species does not represent major difficulties and depends on the fine scale precision of the gene expression annotation generated by the user. However, the existence of different life cycles in marine invertebrates within the same phylum (for example echinoids) gives rise to developmental stages (larval stage) within indirect developing species that are absent in direct developers (no larval phase *per se*). These differences make comparison between two species a difficult task, and it gets more complicated as a greater number of species are added to the list. So to avoid matching non-comparable stages in cross-species analysis we decided to compare only broadly accepted parent stages. In table 1, we compare the developmental stages of the cnidarian *Nematostella vectensis *with the acoel flatworm *Convolutriloba longifissura *and classify the various stages that will be used in the comparison algorithm of our database. Obviously, some temporal resolution is lost when reducing development into only six chronological stages shared among invertebrates, so each parent stage is assigned taxon-specific child stages that are used when doing intra-species comparisons of gene expression.

Even more difficult than comparing developmental stages is the comparison of localized gene expression among 'complex' animals that possess taxon-specific structures (for example gill slits or tube feet). In order to overcome the issue of comparing non-homologous structures, we defined parent territories that can be subdivided in species-specific expression domains. In table 2 we show the expression domains defined for the same species as described above (*N.vectensis *and *C. longifissura*) sorted by broad domains by which it can be argued to be homologous between different taxa.

**Figure 1:**
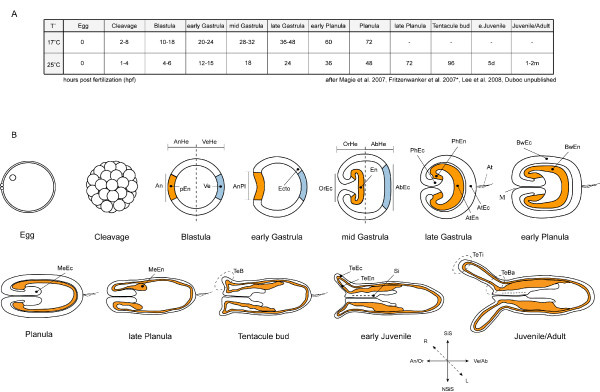
**Chart defining developmental stages and expression domains in *N. vectensis***. (**A**) The twelve main developmental stages of *Nematostella vectensis*, according to [24, 25*, 26], Duboc unpublished, *raised at 18°C) and the developmental time required to reach them (in hours post fertilization (hpf), days (d) or month (m)) depending on the temperature of rearing. (**B**) Schematic representations of the developmental stages indicated in (**A**) with focus on characteristic stage specific anatomical features and a proposed expression domain nomenclature in order to homogenize database entries. All stages are profile views oriented according to the axis key represented at the bottom of the figure. The nomenclature of the later stage replaces its younger equivalent. For example Animal Plate (AnPl) at the early gastrula stage replaces the term Animal pole (An) at the Blastula stage (both presumptive endoderm). (**AbEc**) Aboral Ectoderm, (**AbHe**) Aboral Hemisphere, (**An**) Animal pole, (**AnHe**) Animal Hemisphere, (**An/Or**) Animal/Oral, (**AnPl**) Animal Plate, (**At**) Apical tuft, (**AtEc**) Apical tuft Ectoderm, (**AtEn**) Apical tuft Endoderm, (**BwEc**) Bodywall Ectoderm, (**BwEn**) Bodywall Endoderm, (**En**) Endoderm, (**Ecto**) Ectoderm, (**L**) Left, (**M**) Mouth, (**MeEc**) (Mesentery Ectoderm, (**MeEn**) Mesentery Endoderm, (**NSiS**) Non-Siphonoglyph Side, (**OrEc**) Oral Ectoderm, (**OrHe**) Oral Hemisphere, (**pEn**) presumptive Endoderm, (**PhEc**) Pharyngeal Ectoderm, (**PhEn**) Pharyngeal Endoderm, (**R**) Right, (**Si**) Siphonoglyph, (**SiS**) Siphonoglyph Side, (**TeB**) Tentacle Bud, (**TeBa**) Tentacle Base, (**TeEc**) Tentacle Ectoderm, (**TeEn**) Tentacle Endoderm, (**TeTi**) Tentacle Tip, (**Ve**) Vegetal pole, (**Ve/Ab**), Vegetal/Aboral, (**VeHe**) Vegetal Hemisphere.

To standardize experiments, developmental stages in a given species should correspond to a time line that reflects distinctive developmental stages based on morphological signs and hours of development post fertilization (hpf) at a given temperature that is shared by all users. The same should be done for expression domains, where expression territories at particular times are clearly defined and acknowledged by all users. Figure 1 represents such a chart for *N. vectensis*.

In order to extend this standardization of species-specific developmental stages and expression domains to all species present in the database, we are currently implementing a Comparative Embryonic Developmental database that will provide an overview of the main developmental steps and morphological structures of a variety of animals. We anticipate that this database may help facilitate the move towards a common set of experimental conditions in studies of a particular organism. In addition, by cross referencing both gene expression and embryology databases we can provide information required for the understanding of species-specific development and expression domains for the non-specialist.

#### Adding and editing species, genes and experiments

To get the most use from this database (advanced search, comparison and suggestions of gene expression similarities in a species-specific context) it is important for the user to input stage and expression information in a standardized manner. Therefore, we have made an effort to simplify the upload procedure to the bare minimum that utilizes the user's knowledge of their experimental system. To add a new species (requires registration), the user simply submits an online form, defining the species-specific developmental stages and expression domains associated with the individual developmental stages and expression domains. The user should submit a picture of the adult and a publication relevant to the staging system of the animal. After verification, an administrator adds the species information to the database enabling the user to submit genes and experiments. To add genes and their expression patterns, the user needs to follow the instructions given on the website step-by-step. Mainly, these steps consist of i) adding species-specific gene information (gene name, synonyms (additional and/or former names), and relevant publications if available), ii) assigning an experiment (*in situ *hybridization, IHC) to that gene, providing the required minimum information (type of staining, temperature, vector and so on) that would allow other users to reproduce the experiment, iii) uploading individual images (expression patterns should always be oriented in the defined direction to facilitate comparison), iv) assigning the images to the corresponding developmental stages and v) annotating the expression domains. Each user can edit and delete his/her own experiments and in case a user wishes to edit information not added by him, he can contact the person associated with the information, leave a comment or contact a Kahi Kai administrator. The use of images extracted/cropped from publication figures may fall under copyright infringement of the given journals. We therefore highly recommend the use of original images (that have or have not been used in publications).

A more detailed manual and guidelines can be requested by email or downloaded directly on the website. http://www.kahikai.org/index.php?content=genes.

#### Utility - a case study

In its current state, this database is used to store annotated RNA *in situ *hybridization and IHC information for marine invertebrates that can be searched and compared within or between different species. The expression data is assigned to different experiment types such as wild type expression, mRNA or morpholino injection and so on, making it easy to analyze results from functional studies. We will present the general aspects of the database and guide the reader through the various query options and the analysis of the result pages using as examples published endodermal genes expressed in the cnidarian *N. vectensis*.

#### Querying the database

**Figure 2:**
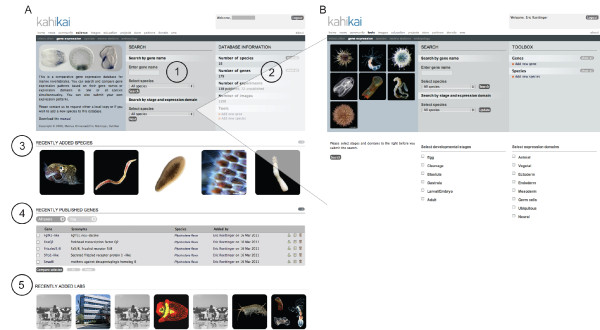
**Database query**. Each panel corresponds to one individual webpage. (**A) **the introduction screen with (1) a search window, (2) general information about the database, (3) recently added species, (4) published genes and (5) laboratories that have contributed gene expression patterns. At this point the user can search a gene expression pattern by entering a single gene name and query in one or all available species simultaneously. (**B) **the advanced search feature for all species (default setting), where the user can search a gene expression pattern by (i) gene name, (ii) developmental stage and/or (iii) expression domain(s). Note that in this case only parent stages (for example gastrula) or parent expression domains (for example ectoderm) are shown for the sake of comparison. If one specific species is selected all sub-stages (for example mid-gastrula) and all sub-domains (for example pharyngeal ectoderm) are shown and can be used for the advanced search (more details about the advance search are shown in **Figure 5**).

The start page (Figure 2) is divided in several parts that give the user a quick overview of general statistics (Figure 2A.2) of recently added species (Figure 2A.3), recently published genes (Figure 2A.4) and laboratories that recently joined the community (Figure 2A.5). The user has two ways of querying the database (Figure 2A.1); i) by entering a gene name in the search field or ii) by using the advanced search feature that allows querying the database by developmental stage and/or expression domain (Figure 2B). Advanced searches allow cross-species searches among all species present in the database by developmental stages and domains to be defined as search values. If the user selects a single species, the entire list of developmental stages and expression domains of that given species would be apparent and could be used to query the database (not shown).

### Query by gene name

#### Species-specific query

**Figure 3:**
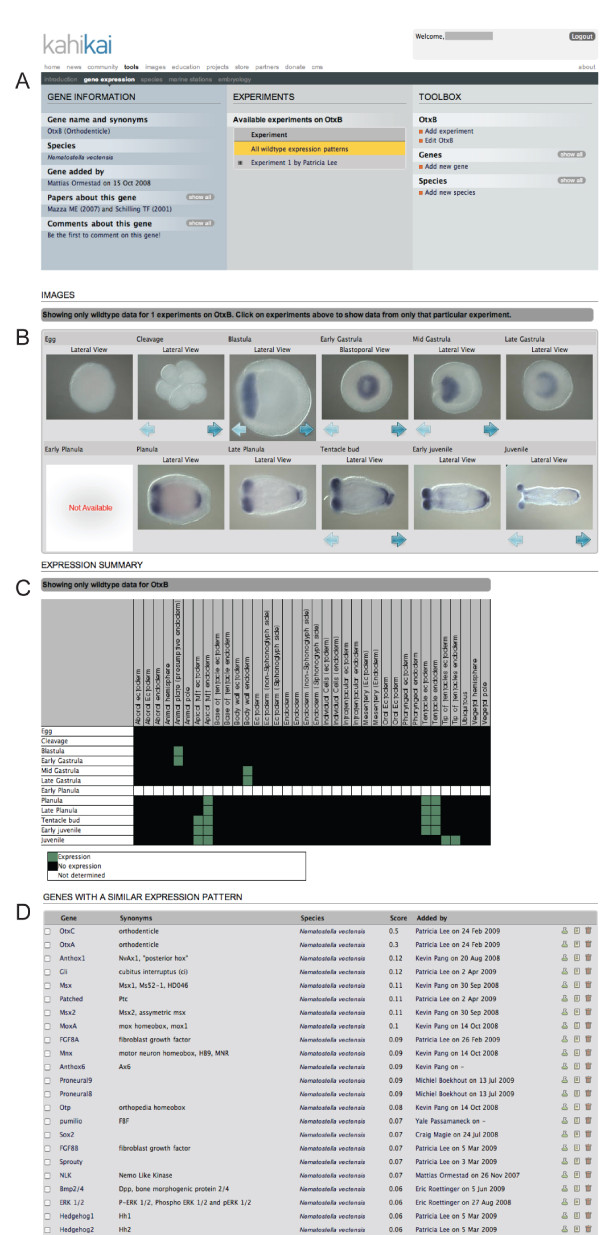
**Result page**. The output for *Nematostella vectensis otxB*. This particular entry shows *in situ *experiments on wild-type embryos. (**A**) General information about gene, species, author, relevant publications and the experiments. (**B**) Image view of the gene expression pattern. Images representing the same stage (same or different view) can be visualized by clicking on the blue arrows at the bottom of each picture. (**C**) This gene expression pattern is also represented in a table view (developmental stages versus expression domain) where the colored cells represent the following information. White: no data available; Black: No expression detected; Green: Expression. (**D**) The software suggests a list of similarly expressed genes.

**Figure 4:**
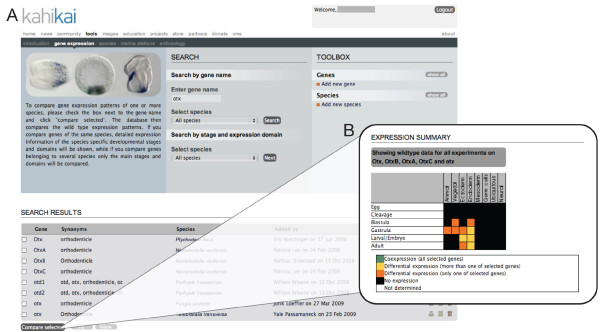
**Cross species query by gene name**. (**A**) In its current state, searching *otx *expression patterns in all species will lead to a list of eight entries; one from *Ptychodera flava *(Hemichordata), three from *Nematostella vectensis *(Cnidria), two from *Parahyle hawaiensis *(Ecdysozoa), one from *Fungia scutaria *(Cnidria) and one from *Terebratalia transversa *(Spiralia). (**B**) The comparison table for all genes reveals the stages in which more than two species share a similar expression domain of *otx*. Clicking on a yellow square will open a new window in which the expression patterns of the given species are shown (similar to **Figure 5**).

**Figure 5:**
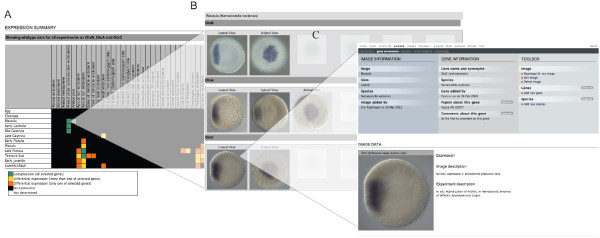
**Species-specific gene expression patterns comparison**, (**A**) The default output of this comparison (in this example *Nematostella vectensis otxA, otxB and otxC*) is a comparison table (developmental stages versus expression domain). Green cells indicate co-expression of the selected genes at a given stage in a given domain, yellow cells indicate that more than one of the selected genes are co-expressed (if more than two expression patterns are compared) and orange cells indicate that only one gene is expressed. Black and white cells indicate no expression or no data available respectively. (**B**) By clicking on a green cell the images corresponding to the three genes sharing an expression pattern at a give stage appear in a new window. (**C**) Final view of a single gene expression pattern showing the gene expression information and experimental data.

The output of a query by gene name, in this example, the *N. vectensis *transcription factor *otxB*, [19] will show general information about gene, species, contributor and relevant publications (Figure 3A left panel). In the center panel the available experiments are listed and the contributor (and affiliation) that uploaded the gene expression pattern. Each registered user can add a new experiment that involves *otxB *expression but only the user that uploaded the selected experiment (in this case Patricia Lee) is authorized to edit information about the experiment, add or delete images and edit the gene expression annotation (stages, view and expression domain). Below (Figure 3B) this general information the expression pattern of *otxB *is shown and is represented by individual images sorted by developmental stages (by default all wild-type expression patterns are shown, including wild-type controls from perturbation experiments). If several images of the same stage (for example different views) have been uploaded, this is indicated by the presence of the blue arrows at the bottom of each image. By clicking on any image, the user will have access to a single image view (example shown in Figure 4C), providing more information about the experiment and the option to access a high-resolution image for personal use or scientific presentations (written permission by the author required). The original paper should be cited and if an unpublished expression is used the author/laboratory and Kahi Kai should be cited. In addition, a table view (Figure 3C) of developmental stages versus expression domain is presented, where the green cells indicate expression of *otxb*. Based on the annotation information provided by each user, the software identifies genes with similar expression patterns (Figure 3D). The similarity score is a percentage of the number of similar stage and/or domain matches in a comparison, and a value of one would correspond to 100% similarity of the annotated gene expression. In our example, *otxC *and *otxA *are the most similar genes to *otxb*. From that proposed list, either an individual gene, several genes or all genes can be selected for comparison with *otxB *(Figure 5A).

#### Cross-species query

In order to compare gene expression patterns across larger evolutionary distances, the user can query the database for all species available in the repository. He can do so by entering the gene name (*otx*) or its synonyms (*orthodenticle, otd*) into the search field on the starting page and keep the species selector on its default position (All species) (Figure 4A). The following results page lists all the *otx *genes from all the species uploaded into the database. In this example, all three above mentioned *N. vectensis *(Cnidaria, [19]), one *Ptychodera flava *(Hemichordata, [20]), two *Parhyale hawaiensis *(Ecdysozoa, [21]), one *Fungia scutaria *(Cnidaria, Loeffler *et al*. unpublished) and one *Terebratalia transversa *(Spiralia, [22] are shown. Clicking the gene name in front of the species name will lead to the entire uploaded expression pattern for that gene in the given animal (data not shown). On the other hand, selecting and comparing all identified genes will lead to a comparison table that will indicate in what animal *otx *orthologs are expressed at a similar developmental stage in a similar expression domain. Clicking the yellow square will provide access to the gene expression patterns in the various species at the given stage. This quick analysis shows that similar to *N. vectenis otxA, otxB *and *otxC, F. scutaria otx *is expressed in the pharyngeal ectoderm in larval stages, and *P. flava otx *is expressed similarly to all *N. vectenis otx *in the presumptive endomesoderm (data not shown).

#### Species-specific comparison of gene expression (*otxA*, *otxB *and *otxC*)

For the following example we selected *otxA *and *otxC *and compared it with the initial *N. vectensis *query *otxB *(Figure 3B). The default output (Figure 5A) of this comparison is a table (developmental stages versus expression domains) in which all green cells indicate co-expression of the selected genes at that particular combination of stage and domain, suggesting that these genes may be involved in a same biological process, defining a putative syn-expression group [16]. Yellow cells indicate that at least two of the three genes are co-expressed at that stage in the given domain, while orange, black or white cells indicate that only one gene is expressed, no gene is expressed or no information is available, respectively. By clicking on one of the green cells the images corresponding to the genes co-expressed at the given stage are shown in a new window (Figure 5B), and by selecting an image, a final window will present detailed information about that particular stage/experiment with the option of downloading the image (Figure 5C). Analysis of these three expression patterns shows that these three cnidarian *otx *paralogs are co-expressed during most of embryonic development, but that otxC is not detected in the apical domain of the planula larvae, suggesting a differential transcriptional control of these factors in later stages (see [19]).

#### Species-specific identification of co-expressed genes

**Figure 6:**
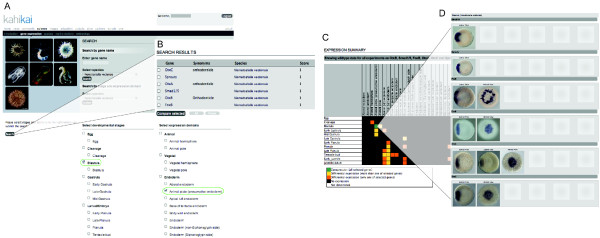
**Species-specific advanced search**. (**A) **A detailed list of all defined developmental stages and expression domains (to reduce the size of the figure some have been omitted) is available for a species-specific advanced search (in this case *Nematostella vectensis*). By selecting the stage(s) and domain(s) of interest (in this example animal plate (presumptive endoderm) at the blastula stage, indicated by green circles) the user will retrieve (**B**) a list of genes that fulfill the search criteria. (**C**) Comparison table obtained after selecting all genes of the result list with a score of one. All green cells indicate that indeed the identified genes are co-expressed in the same domain at the same stage, while their expressions differ in other stages (orange and yellow cells). (**D**) Clicking on the green cell (in this case the blastula stage), will open a new window in which all blastula stages of the given genes are shown allowing the user to verify the information directly on the submitted images.

The previous example demonstrated that all three *otx *genes in *N. vectensis *are co-expressed at the blastula stage in the presumptive endoderm (animal plate). To further understand the initiation of endoderm formation in cnidarians, it would be useful to identify other genes co-expressed with the *otx *genes at the blastula stage. Selecting blastula stage and animal plate (presumptive endoderm) in the advanced search option for *N. vectensis *(Figure 6A) will result in a list of genes that share those properties (Figure 6B). By selecting and comparing all genes with a high similarity score (corresponding to the query) the resulting comparison table (Figure 6C) indicates in green that indeed all the selected genes are co-expressed at the blastula stage while their expression patterns differ in other stages suggesting different transcriptional controls and additional roles of those genes in a different developmental context. Clicking on the green cell will open a new window (Figure 6D) in which all blastula stages of the identified genes are shown for further examination.

#### Cross-species (Larval/Embryo, Ectoderm)

By using the advanced search function on all species (default value) but defining the simplified and optimized 'parent' stage (Larval/Embryo) and the domain (Ectoderm) as search values the user will obtain a list of genes from all species that fulfill the query requirements. The steps are similar to the ones described for the species-specific advanced search and will lead to a comparison table that allow the visualization of the given gene expression patterns with access to a more detailed species-specific annotation simply by clicking on the image of interest. This can quickly identify candidate genes from different species that are expressed with similar developmental patterns.

#### Functional experiments

**Figure 7:**
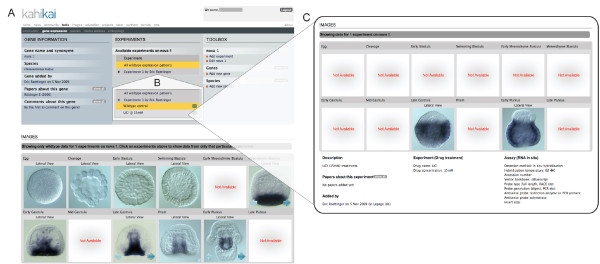
**Organizing functional data**. (**A**) Per default, a compilation of all wild-type expression patterns (indicated by the yellow bar) is shown in the result page (in this example *Paracentrotus lividus nova1*). If selecting the Experiment button in (**A**) the details of the given experiment are un-collapsed (**B**) offering the option to select between the control (wild-type), or the experimental condition (treatment, injection). In a new window the user will then see the *in situ *hybridization carried out on treated embryos with the gene of interest including the experimental details (LiCl 15 mM in this case).

The design of our database not only allows storing and analyzing wild type experiments but also the inclusion of functional studies (Figure 7, drug treatments, RNA or RNAi injection, morpholino, and so on). A compilation of all wild-type expression patterns (wt, control of Experiment 1, control of Experiment 2, and so on) of a given gene (in this example *Paracentrotus lividus nova1*) is shown on the results page, as indicated by a yellow bar (Figure 7A). By selecting one of the experiments listed (in this case experiment 1) details of the experiments are un-collapsed showing the actual functional experiment (LiCl 15mM) and the control it is associated with (Figure 7B). Each functional experiment needs to be linked to a control expression pattern in order to be uploaded. At this stage the user has the option of selecting either the control or the treatment experiment that will open a new window, allowing visualization of all images and associated information (Figure 7C).

## Discussion and future directions

The major challenges for data repositories include the initial and continuous input of data into the database and their long-term sustainability. As described by Merali and Giles [23] community-based and driven databases are generally more successful than projects initiated and maintained by single labs or small research groups. The Evo-Devo field is an interactive and growing community and members are invited to submit their gene expression patterns as well as suggestions for improvement to this new database. Currently, the database contains only data from marine invertebrates, but contributions for terrestrial organisms are welcome. Our hope is that this will encourage researchers to share their data with the Evo-Devo community through a community platform that acts in parallel to peer-to-peer publications, improves the visibility of the published work and fosters scientific interactions.

We hope that community driven projects like this one will help improve the way we publish gene expression data today. Instead of collecting all information in static PDF's for print, all image data should primarily be annotated and published in a searchable and standardized format that then can be summarized for print (with automatic creation of hyperlinks to the online data).

The Kahi Kai non-profit organization is in the process of assembling an international scientific committee from different laboratories. We are considering using part of this committee to screen all added genes to minimize erroneous additions to the database in regard to naming conventions, duplicate entries, orthologies and image orientations. This review process will require all new additions to be put in a queue causing a slight delay (the reviewing time) before the expression data is visible to all users.

To make this tool even more useful, we are planning to add additional features including possibilities to implement qPCR and other quantified expression data such as RNA-seq and microarray that will enable gene regulatory network predictions. Therefore, we will develop tools to organize this information in gene regulatory networks where each node can be linked to the corresponding data, making it easy to check, confirm and compare relations.

Gene expression patterns are defined by precise developmental stages and embryonic regions/or germ layers of a given species. This information is usually only known by specialists, making comparison between species for non-specialists sometimes difficult. To facilitate this, we will associate each species present in the database with detailed information about their embryonic/larval development (database in progress). In addition, we anticipate adding illustrations of the developmental stages high-lighting the various domains relevant for the gene expression data (refer to Figure 1). We also consider that each species present in the database will be associated with information about habitat, life cycle, feeding behavior, spawning season and advice for laboratory cultures. This information will also be illustrated with high-resolution pictures of the animals, which can be used for outreach, education, scientific presentations and publications for example.

We encourage independent Principal Investigators who submit data to open access journals such as *EvoDevo *to also submit expression data to the Kahi Kai gene expression database.

## Conclusions

The present comparative gene expression database allows storing, querying and sharing of data not only with the research community, but also in a more restricted way with a group of collaborators or at the level of a single laboratory. In its current state, this tool has been used to track and store the content of gene expression patterns from current and former lab members allowing new studies based on these resources. As described above, a few steps are sufficient to retrieve the expression pattern of a single gene, compare it to genes expressed in a similar way or even identify putative syn-expression groups in order to predict genetic interactions that can be tested with functional experiments. All available information is tightly linked to the user as well as the laboratory he is associated with, ensuring that each user and laboratory gets proper credit for their contributions. We have a strong focus on making simple and intuitive interfaces and we believe that this comparative gene expression pattern database in its current state will be a useful tool for the research community and students interested in zoology, and evolutionary and developmental biology. Further improvements and additions to the existing database in the future will further enhance its usability for the Evo-Devo community. By integrating scientific data, educational material and general information about animals on a community platform we hope to improve scientific outreach as well as provide students and teachers with means to study and to interact directly with the research community.

## Availability and requirements

The database can be accessed at: http://www.kahikai.org/index.php?content=genes.

To query the database no restrictions apply. To add, edit or delete data, the user needs to be logged in.

## Abbreviations list

GRN: gene regulatory network; hpf: hours post fertilization; IHC: immunochemistry; EST: expressed sequence tag.

## Competing interests

The authors declare that they have no competing interests.

## Authors' contributions

ER and MO conceived and designed the database; MO programmed the database; ER, MO and MQM wrote the article. All authors read and approved the final manuscript.
